# On the choice of the best chunk size for the speculative execution of loops

**DOI:** 10.1371/journal.pone.0267602

**Published:** 2022-05-17

**Authors:** Alvaro Estebanez, Diego R. Llanos, David Orden, Belen Palop

**Affiliations:** 1 Dpto. de Informática, Universidad de Valladolid, Valladolid, Spain; 2 Dpto. Física y Matemáticas, Universidad de Alcalá, Alcalá de Henares, Madrid, Spain; 3 Dpto. Didáctica de las Matemáticas, Universidad de Valladolid, Segovia, Spain; Universidade Federal de Sao Joao del-Rei, BRAZIL

## Abstract

Loops are a rich source of parallelism. Unfortunately, many loops cannot be safely parallelized at compile time because the compiler is not able to guarantee that there will be no dependence violations. Thread-Level Speculation (TLS) techniques, either hardware or software-based, allow the parallel execution of non-analyzable loops, issuing the execution of blocks of consecutive iterations (called *chunks*) while a hardware or software monitor ensures that no dependence violations arise. If such a dependence violation occurs, the chunk that was fed with incorrect values is discarded and re-started, in order to consume the correct information. In the speculative execution of non-analyzable loops, it is very important to correctly choose the chunk size, because this choice dramatically affects the performance of the parallel execution. Bigger chunks imply less scheduling overheads, but smaller chunks allow fewer calculations to be discarded in the event of a dependence violation. To find a good chunk size is not a simple task, because loops may present dependencies that cannot be detected at compile time. In this paper, we present a comprehensive evaluation of different scheduling methods to estimate the optimal chunk size in the speculative execution of non-analyzable loops. This evaluation ranges from the simple, classical methods originally devised to achieve load balancing in loops with no dependencies, to methods that make some assumptions on the distribution pattern of dependencies, such as Meseta and Just-in-Time scheduling. We also propose and evaluate a general, more complex method called Moody Scheduling, that does not require a-priori assumptions to achieve the highest performance.

## 1 Introduction

The nature of loops, composed of a fragment of code that is executed several times, makes them natural candidates for automatic parallelization. Ideally, a loop composed of ten iterations could be executed in parallel by ten threads, by assigning each thread a *loop chunk* composed of a single iteration. If we only have two threads, several options arise: From assigning chunks of five consecutive iterations to each thread, to assign chunks of one iteration (or two or three consecutive iterations) to each thread. In the latter case, each thread would ask for a new chunk after processing the one assigned to it. It is important to highlight that, for efficiency reasons, each individual chunk should be composed of *consecutive* iterations, that will be executed sequentially by the same thread.

When aiming to automatically extract parallelism of irregular loops, Thread-Level Speculation (TLS) [1–3] is considered the most cost-effective technique. TLS techniques aim to execute in parallel the loops whose analysis at compile time is not possible. To do so, TLS relies on a mechanism, which can be hardware or software, in order to ensure that the shared data are accessed by all threads without violating the semantics imposed by the original program, that we can call *sequential semantics*.

When we execute concurrently two portions of a code that was originally intended to run sequentially, we always have a thread that executes a portion that was intended to be executed first in terms of sequential semantics, and a thread that executes the code that should be executed later. When both threads works concurrently, we call the former the *predecessor* thread, while the latter is the *successor* thread. If both threads access to independent data, there is no possibility of violating sequential semantics. The problem arises when both threads access the same datum in parallel. Four situations may arise. The first one is when both threads read the same datum. This situation is harmless: The value returned by these two particular reads will be the same, regardless of the read order. The second one arises when both threads write on the same datum. This is called a Write-after-Write (WAW) dependence. The parallelization mechanism should ensure that the write operation carried out by the successor overwrites the previous one. To ensure the correct order in write operations, each thread keeps a local copy of the data modified during the execution of its chunk. When the threads finish their work, the commit operation is carried out in order, from the non-speculative thread to the most-speculative one, thus preserving the most up-to-date copy of each datum [4].

The third type of a dependence arises when a sucessor thread overwrites a datum that has not been read yet by a predecessor. This is called a Write-after-Read (WAR), *anti*, or *backward* dependence [3]. Our speculative framework solves this situation as follows. When a thread needs to read a datum for the first time, it searches for the most recent copy of this datum, from the most-speculative of its predecessors to the non-speculative one. Once found, the datum is *forwarded* to its own local copy. If none of its predecessors have used this datum so far, then the thread retrieves the reference copy of the datum. For example, suppose that we have four threads, being Thread 1 the non-speculative and Thread 4 the most-speculative. If Thread 3 needs a particular datum for the first time, it searches for this datum in the local data of Thread 2 (the most speculative one of Thread 3’s predecessors). If the datum is found, then Thread 3 forwards a copy in its local data. Otherwise, it searches for the datum in the local data of Thread 1. If none of its predecessors has a copy of this datum, then the reference value is retrieved. The main advantage of our speculative framework is that it handles WAR and RAW dependencies without the need of critical sections, still avoiding race conditions [4].

The fourth type of a dependence is the Read-After-Write (RAW) dependence, also known as *true* dependence [3]. Whenever some thread accesses a datum that was not generated by a predecessor yet, a RAW dependence violation arises. RAW dependences are detected by our framework as follows. When a thread generates a new version of a speculative datum, the thread checks whether any thread *more speculative than it* has consumed a now-outdated value of this datum. If such an offending thread appears, the speculative framework should discard its calculations. Different ideas have been proposed to deal with this issue. The first software-only speculative solutions like [1, 2] opted for interrupting the speculative execution and serially re-executing the affected loop. Later approaches, e.g., [4–6] suggested to squash just the offending thread and its successors, and then re-start them using the correct values for the data. A finer idea appeared in works like [7–9], which also squash only the offending thread as in the previous ones, but in addition squash only the subsequent threads that indeed consumed any value from that offending thread.

One can plausibly realize that TLS is badly affected by frequent squashes. A possibility for mitigating the cost of a squash can be reducing the size of the subsets of iterations (chunks) which are assigned to the threads. On one hand, this reduces the amount of work which would get discarded when performing a squash. On the other hand, this also reduces the probability of appearance of dependence violations. Nonetheless, using smaller chunks also has drawbacks, implying that commit operations would have to be used more frequently and that the scheduling overhead would be higher. Although our framework mitigates the cost of the commit operation by overlapping the commit of the data generated by threads that have already finished with the speculative execution of later chunks [4], choosing an appropriate size of the chunk critically affects the speculation performance.

Several methods have been proposed in previous literature to deal with loops that present dependencies among iterations. These methods not only aim to achieve load balance, but also try to avoid the cost of squashing and re-executing chunks of iterations. These methods range from purely static methods devised to schedule loops with a certain pattern of dependencies, to dynamic methods that monitorize the execution to adjust the size of the following chunk to be issued.

In this work we perform a comprehensive comparison of different methods presented so far, from such simple, classical approaches as Fixed-Size Chunking, to our Moody Scheduling, a sophisticated method that takes into account at runtime both the number of dependence violations and their tendency, so as to adjust the chunk size accordingly.

The organization of the rest of the paper is the following: Section 2 offers a brief overview of software-based, Thread-Level Speculation. Section 3 reviews the classical scheduling alternatives, designed to improve load balancing in the parallelization of loops with no dependencies. Section 4 discusses the peculiarities of scheduling iterations under TLS. Section 5 briefly describes Meseta, a TLS scheduling mechanism focused on extracting the performance from Randomized Incremental algorithms, where the general dependencies pattern is known in advance. Section 6 shows how the re-execution information available at runtime can be used to issue chunks of iterations with a more appropriate size in the general case, and describes Just-in-Time scheduling, the first method that followed this path. Section 7 describes our Moody Scheduling approach, an advanced method that does not only takes into account the number of dependence violations but also their tendency, allowing even more informed scheduling decisions to be taken. Section 8 gives some experimental results, comparing all the evaluated approaches, while Sect. 9 concludes this paper.

## 2 Thread-level speculation in a nutshell

Thread-Level Speculation (TLS) [3], also called Speculative parallelization or Optimistic Parallelization [10], assumes that sequential code can be optimistically executed in parallel, and relies on a runtime monitor to ensure that no dependence violations are produced. A dependence violation arises if a given thread generates a datum that has already been consumed by a successor in the original sequential order. In this case, the results calculated so far by the successor (called the offending thread) are not valid and should be discarded. Early proposals [1, 2] stop the parallel execution and restart the loop serially. Other proposals stop the offending thread and all its successors, re-executing them in parallel (see [5, 11–13]). A third option (see [7–9]) is to restart only the offending thread as well as its successor threads that may have consumed values from it. This approach leads to a noticeable performance improvement in some cases.

Under speculative execution, each thread maintains a version copy of the data structure that is accessed speculatively. At compile time, the original code is augmented to perform speculative stores, speculative loads, and in-order commits. In addition, the loop structure is rearranged to allow the re-execution of squashed iterations. The following paragraphs describe these operations in more detail.

### Speculative stores

All writes to shared data structures should be replaced at compile time with a *speculative store* function. This function is responsible of writing the value to the local copy of the shared variable maintained by the current thread. After that, this function should check the corresponding local versions of threads that are executing subsequent chunks of iterations, in order to detect potential uses of the now-outdated value. If such a situation (called “dependence violation”) is detected, the offending thread and its successors should be restarted, in order to let them consume the updated value.

### Speculative loads

All reads to shared data structures should be replaced at compile time with a *speculative load* function. This function is responsible of obtaining the most updated value available for the element being accessed. To do so, this function first scans the local copies of data maintained by threads that are concurrently executing previous chunks of iterations. If a version of this datum is found, then its value is *forwarded* to the current chunk. If the value has not been used in the previous chunks of iterations, then the value from the reference copy is retrieved.

### Commit-or-discard operation

When the thread finishes the execution of the current chunk, if it has not incurred in a dependence violation due to the use of polluted data, its local changes to the shared variables should be *committed* to their main copies. Note that all threads that have finished should perform this commit operation according to sequential semantics. If the thread discovers that it has been squashed due to the use of a datum that was later changed by a predecessor thread, it simply discards all its calculations, in a so-called *squash* operation. In both cases, after finishing, the thread receives a new chunk of iterations to proceed with the parallel execution of the loop.

## 3 Classical scheduling alternatives for fully parallel loops

The problem of scheduling iterations of irregular loops in order to assign them to different processors has been extensively studied in the literature. An *irregular* loop is a loop whose RAW or WAR dependencies among iterations are not known at compile time, and therefore the loop can not be safely parallelized by the compiler. An irregular loop may or may not present dependencies. As we will see, all classical proposals assume that there are no dependencies among iterations, and therefore all the iterations can be executed in parallel in any order. In this section we review some of the main solutions that have been proposed to this problem. A more detailed description of these solutions can be found in [14].

We first describe the three best known techniques to distribute iterations among processors. Let us call *N* the total number of iterations, and let *P* be the total number of threads (equal to the number of processors in the system). The first, called *static scheduling*, divides the iteration space statically into *N*/*P* chunks of equal size. This system does nothing to balance the workload during the execution of the loops. Hence, the processors may finish at very different times, leading in this case to a poor load balance. However, static scheduling can be a good choice in cases when the target loop exhibits a regular behavior, with all iterations consuming roughly the same execution time. On the other hand, *self scheduling* [15] and *dynamic scheduling* use different chunk sizes as the execution progresses. The main difference between *self* and *dynamic* scheduling is that *self scheduling* defines chunk sizes before the execution starts, while *dynamic scheduling* mechanisms adjust chunk sizes at runtime. These approaches minimize load imbalance, but at the cost of greater overheads, because smaller chunks imply more frequent scheduling and commit operations.

It is worthwhile noting that none of these methods takes into account the possibility of a dependence violation. Both *static* and *dynamic* scheduling methods are offered as part of the *schedule* clause present in OpenMP.

Within self scheduling, different alternatives have been proposed. A brief description follows.

### Fixed-size chunking (FSC)

In this approach [16], the iteration space is statically divided into chunks of fixed size. Each free thread executes the following chunk. This solution reduces the synchronization overhead in comparison with issuing a single iteration each time, with a better load balance than static scheduling. Choosing an appropriate value for the chunk size, *K*, is critical for this scheme to be efficient. Such a choice is difficult and can only be carried out with extensive experimentation.

### Guided self-scheduling (GSS)

This technique, proposed by Polychronopoulos and Kuck [17], addresses the problem of uneven start times for each processor. Instead of using a fixed chunk size, they propose decreasing chunk sizes, calculated as a decreasing function of the current iteration number *i* being executed. As execution proceeds, smaller chunks improve the balance of the workload toward the end of the loop.

In order to avoid having many small chunks by the end of the loop, an additional function GSS(*K*) is proposed to bound the chunk size from below by *K*, specified either by the compiler or the programmer.

Wang *et al*. [18] developed a version of GSS intended to mitigate hardware faults on shared memory systems.

### Factoring

This mechanism, proposed by Hummel *et al*. [19], is similar in concept to guided self-scheduling, but the allocation of iterations to processors proceeds in phases. In each phase, a part of the remaining iterations is divided into batches of *P* equal-size chunks.

Factoring can be viewed as a generalization of GSS and Fixed-Size Chunking: GSS is factoring where each batch contains a single chunk, while Fixed-Size Chunking is factoring with a single batch.

### Trapezoidal scheduling (TSS)

This technique, proposed by Tzen and Ni [20], uses chunks that decrease in size linearly. This approach is simpler to implement than GSS and especially GSS(*K*), thus reducing scheduling overheads. Moreover, according to their authors, a big value of *K* in GSS(*K*) leads to a high imbalance, while small values lead to too much scheduling overhead. Consequently, an optimum value of *K* for GSS is difficult to obtain, particularly in unbalanced loops. By decreasing the chunk size linearly, TSS reduces the number of chunks, and hence the overhead, while simplifying the calculation of the next chunk size, allowing its computation with atomic *Fetch-&-Increment* operations.

If the loop being speculatively executed is composed of *N* iterations, the total number of iterations being scheduled is at least *N* for all scheduling alternatives described. However, only the Self Scheduling method guarantees that exactly *N* iterations are issued. Other methods may attempt to issue more than *N* iterations. Consequently, the scheduler should always check whether the upper limit of *N* iterations can be exceeded and order the execution of only the remaining iterations, without issuing the execution of iterations outside the iteration space. The same is true if the total number *N* of iterations is unknown until full completion of the execution.

We now describe some dynamic proposals, that determine the optimum chunk size at runtime based on the total available parallelism, the optimal grain size, and the statistical variance of execution times for individual tasks.

The Tapering algorithm by Lucco [21] was one of the first dynamic approaches centered on augmenting GSS methods with runtime parameters. Markatos and LeBlanc [22] proposed the affinity scheduling, in which iterations were mainly assigned taking processor affinity into account. Iterations were divided into the available processors until load imbalance occurred, when idle processors ‘stole’ some iterations from others. Later, Jin *et al*. [23] improved this algorithm by allowing the number of iterations to execute in each chunk to be changed. This was one of the first approaches of pure-dynamic scheduling. Its authors used the number of iterations executed so far to change the sizes of the following chunks. In the initial phase of their scheduling policy, a first set of iterations was equally divided among the available processors. Then, at runtime, some of the remaining iterations of each processor were downloaded to a queue, allowing other processors to execute them after finishing their own work. The number of iterations downloaded changed according to the workload of the processor. Chen and Guo [24] proposed an enhancement of the OpenMP *static* scheduling (based on FSC), introducing dynamic chunk sizes so as to better mitigate load imbalance.

## 4 Scheduling iterations under TLS

Thread-level speculation aims to manage the parallelization of loops even in the presence of dependence violations. Therefore, the scheduling problem in this case is more complex than the scheduling carried out by the classic methods described above. In this section we will highlight the main differences between the scheduling of loops with and without dependencies. A more in-depth analysis can be found in [14].

Under TLS, the execution of an iteration or chunk of iterations can be discarded, so the scheduling method should be able to re-assign the squashed iteration to the same or to a different thread. The loop structure should also be changed to allow the re-execution of iterations [4]. As can be easily seen, frequent dependence violations and the consequent generation of squashes have an adverse effect in the performance.

Some approaches deal with TLS scheduling by addressing the problem of how to schedule threads accordingly (see [13, 25, 26]), or by managing instruction scheduling [27]. Instead, we center our discussion on how to calculate the size of the next chunk to be assigned to a processor, an issue that directly affects TLS performance.

The simplest solution is to apply the Fixed Size Chunking method described in the previous section. Its main drawback is that it is not possible to calculate in advance the right chunk size. To use it, the programmer need to run the code repeteadly, using different chunk sizes for each input set, in order to determine which chunk size is most appropriate for each parallelized loop. This technique leads to good results if the parallel execution of the loop does not lead to dependence violations. In these cases, the main goals are avoiding scheduling overheads as much as possible and achieving a good load balance in the execution of the last chunks. Uses of this technique can be found in [28, 29].

TLS papers usually give only brief details of their scheduling policy, e.g., [30], and many do not describe their selected mechanism to schedule iterations. Thus, we suppose that most works use FSC-based approaches, many of them delivering chunks of a single iteration. For example, Gupta and Nim [1] affirm that their classic solution could easily be enhanced with a dynamic scheduler of iterations, but they did not give more details. Raman *et al*. [31] gave some hints about a load balancing algorithm which dynamically assigned iterations, but again details are scarce. Feng *et al*. [32], in their approach, focused on adapting I/O operations to TLS approaches, using the GSS algorithm described in the previous section.

There are also some solutions based on dependence analysis at compile time [25, 27, 33]. Such approaches review the possible dependence patterns in order to take scheduling decisions, thus needing of an in-depth analysis of the loop.

Other solutions take advantage of the *expected* dependence pattern of the loop they want to parallelize. A prominent example are Randomized Incremental Algorithms, which are known to show a pattern where the first iterations of the loop accumulate the largest part of the dependencies. In order to improve the performance of Randomized Incremental Algorithms, two methods were proposed. Meseta [34], opts for dividing the execution into three phases. An initial stage, until reaching certain lower bound for the probability of a dependence arising, schedules chunks of increasing sizes with the aim of compensating for possible dependence violations. Afterwards, a second stage consists in applying FSC to execute the major part of the iterations remaining. Finally, at a third stage the chunk size is gradually decreased, so that a better load balance is aimed. An alternative mechanism called Just-In-Time (JIT) scheduling was proposed in [35] which, on one hand, defines several logarithmic functions in order to issue chunks of increasing size and, on the other hand, uses runtime information in order to modulate those logarithmic functions taking into account how many dependence violations effectively appear. Both Meseta and Just-In-Time scheduling methods will be described later in more detail, in order to better understand the experimental results.

Kulkarni *et al*. [10, 36] discussed the importance of choosing the proper abstractions for the data structures in irregular loops, with the aim of facilitating their speculative execution. These authors parametrize scheduling policies using three design choices, namely *clustering*, *labeling*, and *ordering*, to specify how a schedule behaves. Using their Galois framework, the authors tested different strategies for each of the modules defined. The results show that each of the applications they analyzed could be closely linked to one of different scheduling strategies, thus advocating the implementation of application-specific scheduling policies. More recently, Li *et al*. [37] propose AdapTRA, an adaptive thread partitioning approach for irregular programs that analyzes the program and generates a set of candidate partition schemes. A suitable partition scheme is then selected based on a complexity model of the irregular code to be scheduled.

Some approaches [38, 39] use Decoupled Software Pipelining to enhance the scheduling of iterations. This technique, instead of executing full iterations by the same thread, is based on dividing each iteration into smaller parts and assigning them to the available threads. Thus, threads, ordered forming a pipeline, execute parts of all the iterations. Tian *et al*. [40], in their Copy-or-Discard approach, addressed scheduling by unrolling loops to reduce dependencies. Oancea *et al*. [41] tried to schedule iterations whose instructions had dependencies among them to the same processor, i.e., avoiding the sequential order. To do so, they needed to perform a dependencies analysis before parallel executions. [42] introduced TProf, a profiler that targets the T4 compiler [43] on the Swarm architecture that aims to detect points of parallel contention. The use of this tool allows the programmer to schedule parallel tasks to maximize performance.

Both [44, 45] proposed the most similar scheduling techniques to those that are detailed in this article, JIT [35] and Moody [46] (described in Sect. 7). Specifically, they increased or decreased the number of executed iterations regarding the runtime parameters that reflected the number of dependence violations produced.

Finally, other scheduling strategies have recently been proposed for specific domains to handle the speculative execution of their workloads, such as Sched+ [47] to leverage cloud-based computational loads, or Chronos [48] to schedule the speculative execution of MapReduce jobs.

The purpose of this work is to compare different run-time scheduling methods for thread-level speculation. The first task is to choose the methods to be compared. The review of the existing literature with respect to thread-level speculation proposals shows that (1) Fixed-Size Chunking is a common solution as it is simple to implement; (2) few TLS papers give details about the scheduling policy used in their proposals; and (3) some scheduling solutions for TLS rely on a prior compile-time analysis, or the use of application-specific scheduling policies that are closely related to the data structures used in the sequential implementation. Both techniques fall outside the scope of this work. With respect to the popular, classical scheduling solutions described in Sect. 3, all of them deal with the problem of load balancing by assuming that the loop is completely parallelizable, and that each iteration will be executed exactly once. This assumption makes these scheduling techniques useless when dealing with the parallel execution of loops whose chunks of iterations may be squashed and restarted several times.

In this paper we compare the use of Fixed Size Chunking in the context of TLS with the only methods that, to the best of our knowledge, can be applied with little or no knowledge of the actual dependency pattern of the loop speculatively executed. These methods are Meseta, a simple strategy that is an adaptation of Trapezoidal Self-Scheduling; JIT Scheduling, a dynamic/adaptive solution that was the first that adjusts the chunk size at runtime in response to dependence violations; and Moody Scheduling, a more general solution that makes no assumptions about the actual dependence pattern. We first explain these methods in detail.

## 5 Meseta: Scheduling strategy for randomized incremental algorithms

Meseta [34, 49], a scheduling strategy for TLS based on Trapezoidal Self Scheduling, was designed to deal with loops where most of the dependence violations are located in the first iterations of the loop. This is indeed the case of Randomized Incremental algorithms, an important class of algorithms that obtain an acceptable average execution time by randomizing the input set before being processed. This strategy, widely used in the field of Computational Geometry, allows a solution to be gradually constructed, by iteratively processing the input set at random. While the first points processed are likely to change the current solution, changes are less probable as the computation advances. Therefore, dependence violations are far more frequent at the early stages of a loop execution than at the end. Since a particular dependence violation only leads to a squash if it crosses a chunk boundary, this fact complicates the choice of an optimum chunk size in FSC, because the number of squashes is not known until execution time and it depends on the size of the chunks.

**Fig 1:**
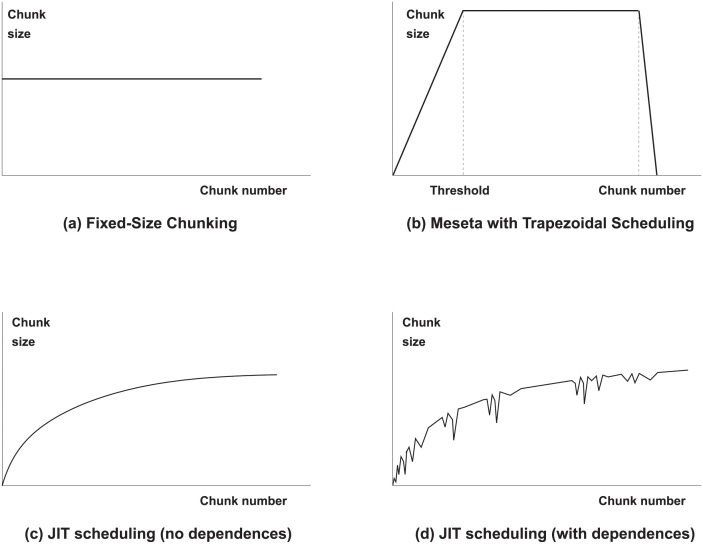
Plot of the chunk size depending on the chunk number with different scheduling strategies: (a) FSC, (b) Meseta scheduling following the trapezoidal approach [20]. Finally, (c) and (d) show Just-In-Time scheduling in loops with and without dependencies, respectively.

The general strategy followed by Meseta is to divide the loop execution into three parts. (see Fig 1(b)). The first part schedules chunks of increasing size as execution proceeds, with the aim of mitigating the adverse effects of the high number of dependence violations at the beginning of the loop. The second part assumes that, after a certain threshold, the number of dependence violations will be stable. Most of the computation is carried out in this part. Finally, the chunk sizes are progressively reduced, in order to achieve a good load balance among the collaborating threads, where any of the techniques proposed in Section 3 can be applied.

Meseta requires the user to set three parameters before being used. The first is to determine the number of iterations that should be executed in the first stage, when the probability of finding dependence violations is still very high. Fortunately, accuracy at this point is not critical for the success of the scheduling mechanism, which performs well regardless of the particular transition point chosen. A second, more important decision is to fix the chunk size for the stable part of the loop. This decision depends not only on the dependence pattern, but also on the overheads produced by squashed threads. Therefore, it is highly dependent on the given problem, and its value should be set through experimentation. Finally, we should also set the percentage of iterations scheduled for the third part. This value, in turn, allows the iteration number in which the descending part of Meseta starts to be determined.

While Meseta is a better alternative than FSC for Randomized Incremental algorithms, its usefulness depends on the particular pattern of dependence violations that can be observed in this family of algorithms.

## 6 Just-In-Time scheduling

To the best of our knowledge, JIT scheduling [35] was the first scheduling method which proposed a solution for scheduling loops whose iterations have dependencies among them without requiring either an in-depth knowledge of the problem, and/or a number of dry-run experiments to learn how to schedule iterations. In this section we briefly describe Just-In-Time scheduling, an approach that adapts its behavior automatically using runtime parameters involved with the occurrence of dependencies.

In order to compute the number of iterations to be scheduled for the next thread, let *e*(*h*) be the amount of times the previous *h* chunks were executed, considering not only the successful executions, but also the re-executions needed due to dependence violations. Note we are supposing all iterations to have the same complexity for clearness. Hence *e*(*h*) = *h* if no dependence violations among threads have arisen because no chunk needed to be re-executed. If we take the average number of executions per chunk $\bar{e}\left(h\right)=\frac{e(h)}{h}$ as a general guide, a scheduling mechanism may adapt the number of chunks based on the number of dependence violations. Thus, it may issue bigger or smaller chunks if no re-executions are needed, or if there are dependence violations in the last chunks respectively.

At the beginning (compile time) the number of re-executions is unknown, however, we can define a set of execution counters so as to save the amounts for each scheduled chunk. As a result, a thread starting its execution can take advantage of $\bar{e}\left(h\right)$ to compute its own chunk size.

These counters are not the only data taken into account to compute chunk sizes. The total number of iterations *N* is a critical datum: Imagine our process has ten dedicated processors. For loops of millions of iterations, a chunk of ten thousand iterations is completely reasonable. Nonetheless, if the loop has only fifteen thousand iterations and we use the same number of iterations, it will lead to a severe load imbalance. Similarly, the index *i* of the first iteration of the chunk to be scheduled is a crucial datum. The appearance of dependence violations is completely random, and therefore, it makes sense to start with small chunks and adjust their size using the runtime information as execution proceeds.

The JIT scheduling method considers the three values mentioned as follows: $\bar{e}\left(h\right),N$ and *i*. The thread responsible of executing the following chunk will calculate the chunk size *C* using the information provided by the three variables mentioned. It depends directly on *N* and *i* and inversely proportional to $\bar{e}\left(h\right)$. This work proposes this formula in order to calculate the chunk size

$$\begin{array}{c} C=\lceil \frac{\;\text{ln}(i)\cdot \;\text{ln}(N)}{\bar{e}\left(h\right)}\rceil \end{array}$$ (1)

Note if the execution does not lead to dependence violations at runtime in the last *h* chunks, both the last *t* execution counters, and $\bar{e}\left(h\right)$ will be equal to one. Fig 1(c) shows graphically how the function would look like in this example. However, if any dependence has arisen during the execution of the last *t* chunks, their execution counters will be higher than one. As a result, the value of $\bar{e}\left(h\right)$ will as well be greater than one, making the size of the following chunk smaller than another execution without dependencies. In the general case, the number of squashes decrease, and therefore, the scheduling function will present some “potholes”, as shown in Fig 1(d). In order to initialize the value of *h* we recommend to use a fixed or proportional value to the number of processors. The usage of a logarithmic function in both *N* and *i* allows to smooth the increase or decrease in the chunk size.

As a result, if the iterations do not contain any dependence, Eq 1 will increase chunk sizes. This fact conflicts with the desire to obtain a good load balance towards the end of the loop, although avoiding the performance degradation due to misspeculation is more important for speculation performance.

The function given by Eq 1 delivers small chunk sizes, a desirable situation if the loop has up to a few thousand iterations. For example, if we consider a loop of 10000 iterations and without dependencies, the biggest feasible chunk size at the end of the loop (say, at iteration 9900) would be *C* = ⌈ln(9900). ln(10000)⌉ = 85 iterations. In the same way, if a loop would have ten million iterations, the maximum chunk size attainable value of *C* would have been just 260 iterations. The usage of chunks with such small number of iterations would increase the execution overheads. So, for such loops, the authors suggested a new formula that let *C* grow faster:

$$\begin{array}{c} C=\lceil \frac{\;\text{ln}{\left(i\right)}^{2}\cdot \;\text{ln}\left(N\right)}{\bar{e}\left(h\right)}\rceil \end{array}$$ (2)

Following the examples mentioned before, the biggest value for *C* produced by Eq 2 in a loop with ten million iterations is 4187 iterations, thus reducing the execution overheads.

Finally, it is interesting to note that any base for the logarithmic function used in Eqs 1 and 2 will lead to similar behaviors as in the use of ln(*x*). In our experiments, we have used ln(*x*) because the cost of its calculation was small enough, representing less than 0.01% of the experiments’ execution time.

### 6.1 Dynamic and adaptive implementations

**Fig 2:**
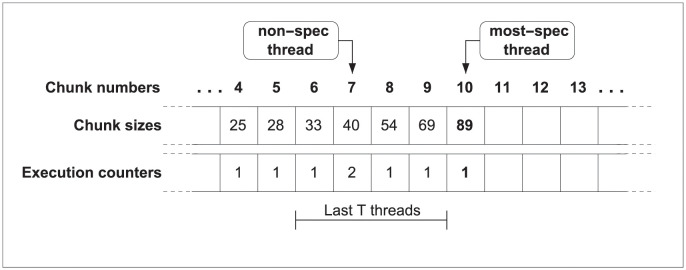
JIT scheduling, dynamic approach. The first time chunk 10 is executed, its size is calculated using the JIT scheduling function and its execution counter is set to 1. The size will be preserved regardless of the number of re-executions of chunk 10.

With the scheduling method described above each thread computes the number of iterations ahead of starting the execution. At that time, the thread that starts the execution of a new chunk will be the most speculative one, being able to access to the execution counters of all its predecessors. Then, the most-speculative thread will set to one its own execution counter, calculate the chunk size, and start the execution of its chunk of iterations, beginning with the next iteration of the preceding chunk. To avoid race conditions with other threads, reads and updates of the execution counters are performed inside a critical section. Fig 2 details this information.

If our thread is squashed, it is because some predecessor has detected a dependence violation and issued a squash event to all its successors. We can address this issue through two ways. As first and simpler solution, we can reuse the same chunk size already calculated for this thread. Since we use a critical section to calculate the chunk size, avoiding to recompute a value allows to reduce the execution time. Obviously, the counter for the number of executions of threads is useless with this solution because the chunk size is only computed once, being one always the value of this counter at that time.

However, with this simple approach we are omitting the significant difference between the raw calculations and the numbers adapted due to an eventual squash operation. So, if a dependence violation arises and some threads have to be re-executed, only new threads, which computes the value of the new chunk for the first time, will take this situation into account, using the new values of the counters. While threads squashed will restart their execution with the same parameters.

**Fig 3:**
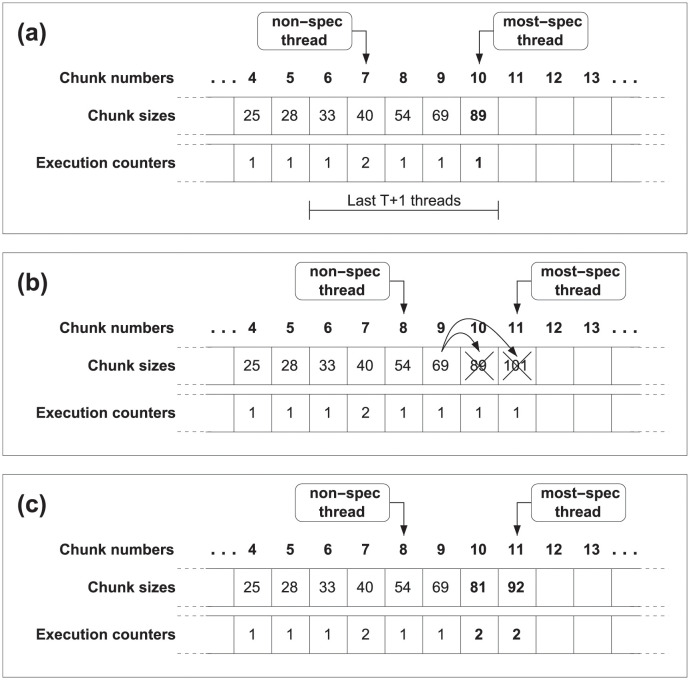
JIT scheduling, adaptive approach. (a) Size of chunk 10 is calculated using the JIT scheduling function. (b) Chunk 9 issues a squash operation. (c) Squashed threads recalculate in program order the size of the chunk to be executed, using the new value of the execution counters.

The second solution focuses on taking into account these squashes which change the execution counters, and simply proposes to compute again the chunk size of the squashed threads before restarting the execution. We can see this behavior in the Fig 3. Therefore, we check *t* + 1 execution counters because we not only take into account the execution counters of the squashed threads, but also the counter from current chunk. This process, detailed in the following section, is more accurate in regard to the events occurred at runtime, but, as more calculations are needed, it adds some overhead.

In summary, if the parallel execution does not lead to dependence violations, the size of the following chunk will be computed only when the thread is going to start its execution. Contrarily, if any dependence arises, the scheduling method allows to modify chunk sizes with the updated and more accurate information. At this point, there are two options. Either to compute the size of the following chunk only the first time this particular chunk is issued or to re-calculate the size of the following chunk each time the chunk is scheduled. The first approach, called *dynamic scheduling* (see Fig 2), preserves the same size for subsequent re-executions. The second solution, called *adaptive scheduling* (see Fig 3) tailors the size for subsequent re-executions.

The advantage of adaptive over dynamic scheduling is that the first computation of the chunk size may use obsolete data because there may be squashes during the execution of the predecessors of a chunk. Adaptive scheduling will have always accurate numbers as it takes into account all the updates in the counters. These constant updates, however, require to call more times to the scheduling function with the costs associated with it.

## 7 Moody scheduling: Philosophy and design guidelines

In [14, 46] we proposed Moody Scheduling, a scheduling method which tries to predict, at runtime and without the need for any knowledge about the underlying problem, the best size for the following chunk using a scheduling function. A description of the method follows. A more detailed description can be found in [14].

**Fig 4:**
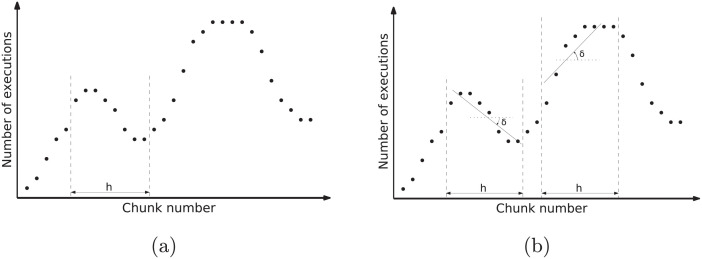
(a) A possible execution profile for a given loop, and (b) an example of the use of linear regression to measure the tendency of the last *h* chunks. Recall that the *y*-axis does not represent the chunk size, but the number of re-executions for each chunk.

Moody Scheduling takes into account the number of squashes and re-executions needed for the last *h* chunks so as to determine the size of the next chunk to be scheduled. Fig 4(a) presents an example where we can compare for each scheduled chunk (*x*-axis), the number of times it has been executed so far (*y*-axis).

This scheduling method calculates the size of the following chunk to be scheduled based on two parameters: $\bar{\text{e}}(\text{h})$ and *d*. They use the information of the last *h* chunks regardless of whether they had already been committed or not. On the one hand, the $\bar{\text{e}}(\text{h})$ is the average number of executions of the last *h* chunks, and, as such, its value is always higher or equal to 1. On the other hand, *d* is the *tendency* of these re-executions. Hence, *d* ∈ (−1, 1) and shows whether the number of executions remains unchanged (*d* = 0), is increasing (*d* > 0) or is decreasing (*d* < 0). As we will see, *d* depends on the angle *δ* between the linear regression line for the last *h* chunks and the horizontal axis (see Fig 4(b)).

Before explaining in the following section the implementation details and the mathematical background behind this idea, we introduce a straightforward summary below and in Table 1.

**Table 1:** Changes on the following chunk sized according to *d* and $\bar{\text{e}}(\text{h})$ parameters.

	$\bar{\text{e}}(\text{h})\approx 1$	$\bar{\text{e}}(\text{h})\approx \text{accMeanH}$	$\bar{\text{e}}(\text{h})\approx \text{maxMeanH}$	$\bar{\text{e}}(\text{h})>\text{maxMeanH}$
*d* → −1	↑	↗	=	1
*d* ≈ 0	↗	=	↘	1
*d* → 1	=	↘	1	1

1. *d* close to -1: The tendency of re-executions is decreasing.
  - If $\bar{\text{e}}(\text{h})$ is close to 1 (very low), we optimistically set the chunk size to maxChunkSize (the maximum size appropriate for this algorithm).
  - If $\bar{\text{e}}(\text{h})$ is between the minimum value (1) and accMeanH (an *acceptable* value for the average of re-executions), we optimistically increase the chunk size.
  - If $\bar{\text{e}}(\text{h})$ is between accMeanH and an maxMeanH (an upper limit for the average of re-executions), the chunk size remains unchanged in order to continue reducing $\bar{\text{e}}(\text{h})$.
  - If $\bar{\text{e}}(\text{h})$ is higher than maxMeanH, we use 1 as the size of the following chunk.
2. *d* close to 0: The tendency of re-executions is stable.
  - If $\bar{\text{e}}(\text{h})$ is close to 1 (very low), then we optimistically issue a larger chunk size.
  - If $\bar{\text{e}}(\text{h})$ is close to accMeanH, then the same chunk size remains unchanged.
  - If $\bar{\text{e}}(\text{h})$ is between accMeanH and maxMeanH, then the chunk size is pessimistically decreased.
  - If $\bar{\text{e}}(\text{h})$ is higher than maxMeanH, then the size of the following chunk is set to 1.
3. *d* close to 1: The tendency of re-executions is increasing.
  - If $\bar{\text{e}}(\text{h})$ is close to 1 (very low), then the same chunk size is kept, so as to confirm whether $\bar{\text{e}}(\text{h})$ really gets larger or not.
  - If $\bar{\text{e}}(\text{h})$ is close to accMeanH (acceptable), then the chunk size is decreased in order to reduce the number of executions.
  - If $\bar{\text{e}}(\text{h})$ is close to (or bigger than) maxMeanH, then the chunk size is set to 1 in order to reduce drastically the number of re-executions.

Finally, we need to decide what is the best size to initialize the size of the first chunk, when we do not have any information about the re-executions. As exposed in Section 8, the use of 1 as initial value produces good performances in all the algorithms tested.

Therefore, if we know the current lastChunkSize and a pair of values $\left(d,\bar{\text{e}}(\text{h})\right)$, the scheduling function will follow the principles detailed above to suggest a new value for nextChunkSize. The following section explains more in detail this implementation.

### 7.1 Moody scheduling function definition

The aim of this function is to calculate a new value for nextChunkSize. To do so, we use *d*, $\bar{\text{e}}(\text{h})$ and the current value of lastChunkSize. *δ* is obtained computing the regression line determined by the last *h* points in our execution window (see Fig 4(b)).

The main problem with the procedure detailed above is that it leads to a discontinuous function. The usage of this simple idea, with nested *if…then* constructs, would imply significant differences for very similar cases.

**Fig 5:**
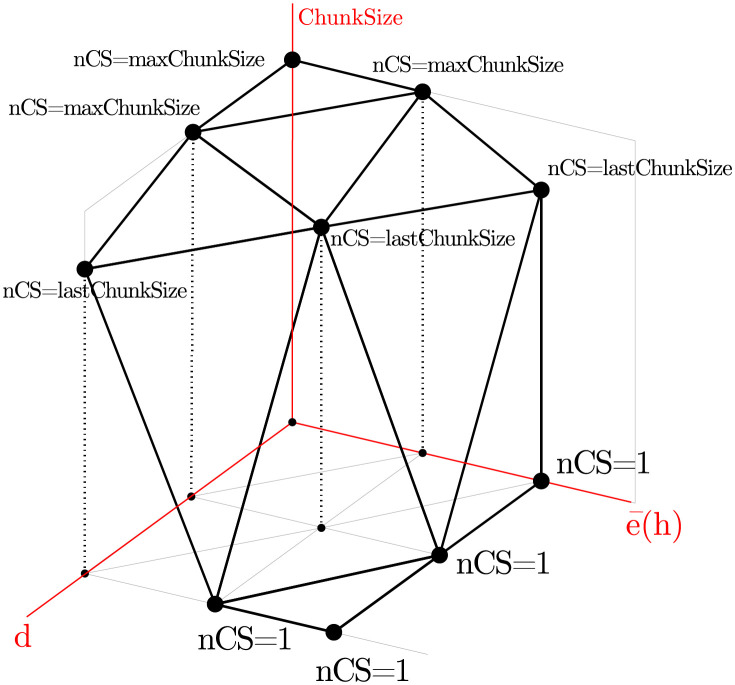
3D representation of the Moody Scheduling function, that returns a value for nextChunkSize (nCS) provided the current lastChunkSize and depending on *d* and $\bar{\text{e}}(\text{h})$.

**Fig 6:**
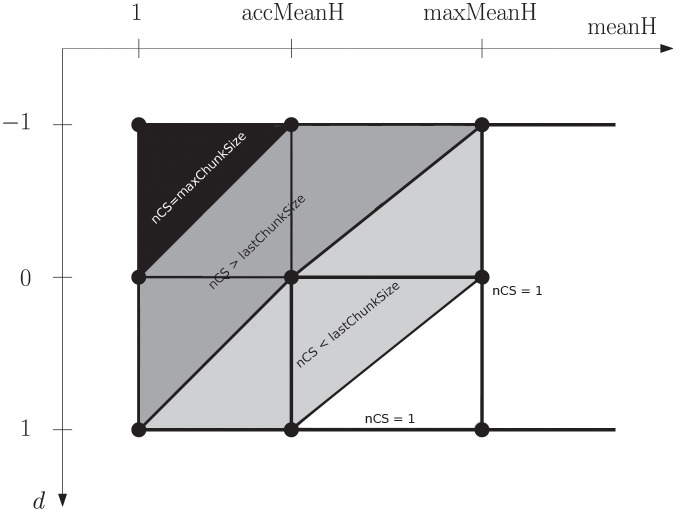
2D representation that connects our function with the intuitive behavior described in Sect. 7.

To deal with that, we define a two-dimensional function that returns the size of the next chunk to be scheduled for a given value of $\bar{\text{e}}(\text{h})$ and *d*. Fig 5 depicts a 3D representation of the Moody Scheduling function proposed. Fig 6 depicts its projection onto a horizontal plane with the same gray scale used in Table 1.

We should define several parameters in order to define this scheduling function properly. *d* is computed measuring the angle *δ* of the tendency with respect to the horizontal axis. This angle lies in (−*π*/2, *π*/2). Our growth tendency *d* ∈ (−1, 1) is given by $d=\frac{\delta }{\pi /2}$.

accMeanH is the highest value of $\bar{\text{e}}(\text{h})$ considered as acceptable. To define this value, we will assume that, on average, chunks have to be re-executed at most once, so we use accMeanH = 2 as initial value.

**Fig 7:**
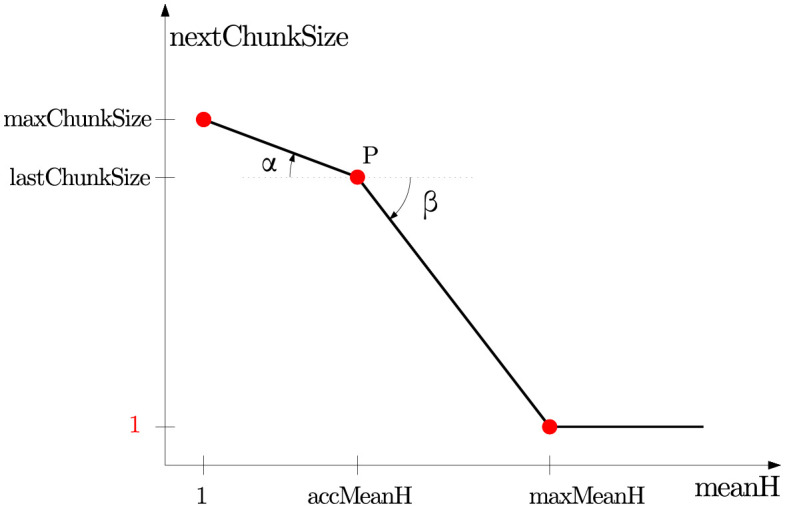
Intersection of the graphic of $\text{nextChunkSize}(d,\bar{\text{e}}(\text{h}))$ with *d* = 0.

maxChunkSize and maxMeanH depends on the slopes of the graphic of the two-dimensional scheduling function as follows. If we fix *d* = 0 in the scheduling function, we obtain two angles, *α* and *β* (see Fig 7). They represent how optimistically or pessimistically the chunk size is going to increase (*α*) or decrease (*β*) respectively. So the bigger the value of the angles, the quicker the size will change. Once the value of the angles is fixed, we obtain the value of maxChunkSize. It is specified as the intersection between the vertical line defined by $\bar{\text{e}}(\text{h})=1$, and the segment from *P* with angle *α*. Similarly, the value for maxMeanH is specified as the intersection between the horizontal line defined by nextChunkSize = 1, and the segment from *P* with angle *β*. If lastChunkSize = 1, then *β* will be 0. In addition, *α* ≠ 0 as long as accMeanH is never set to 1.

The values described above define the nine special points determined by $\bar{\text{e}}(\text{h})\in \{1,\text{accMeanH},\text{maxMeanH}\}$ and *d* ∈ {−1, 0, 1}. Given that the call to $\text{nextChunkSize}(d,\bar{\text{e}}(\text{h}))$ will return maxChunkSize for the three points (−1, 1), (−1, accMeanH), and (0, 1), the function will also return maxChunkSize to all points inside this triangle. Similarly, for all points inside the triangle with vertices (1, accMeanH), (1, maxMeanH), and (0, maxMeanH), the function will return 1. Notice that points on the diagonals (1, 1) to (0, accMeanH), and from there to (−1, maxMeanH) will return lastChunkSize. These three facts provide a natural triangulation for the space in Fig 6.

### 7.2 Dynamic and adaptive implementations

**Fig 8:**
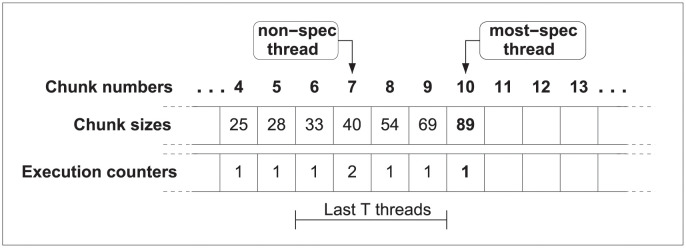
Dynamic Moody Scheduling. The size for the following chunk to be executed (#10 in the example) is calculated once, and its size will be preserved regardless of the number of re-executions of this chunk.

**Fig 9:**
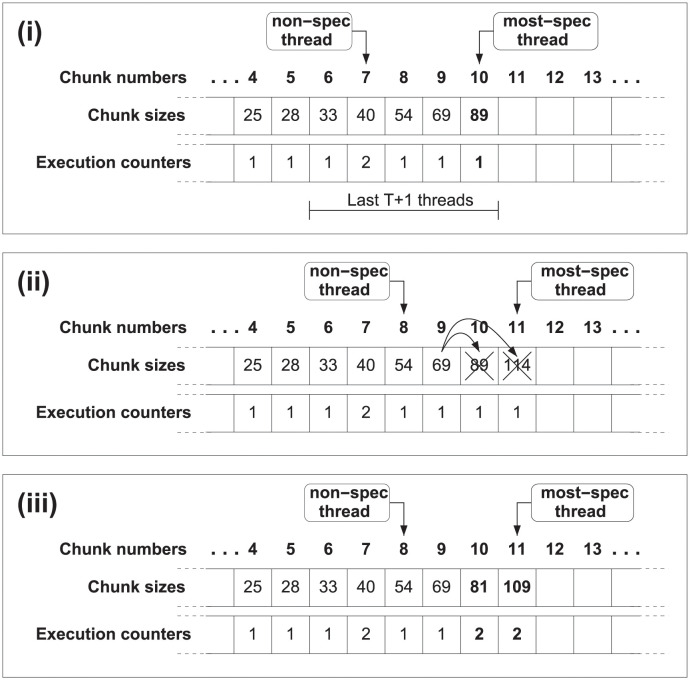
Adaptive Moody Scheduling. (i) Size of chunk #10 is calculated with the Moody Scheduling function. (ii) Chunk #9 issues a squash operation. (iii) All squashed threads recalculate in program order the size of the chunk to be executed, using the updated values of the execution counters.

As it happens in JIT scheduling (see Section 6.1), this approach can also manage both the dynamic and adaptive implementations described above. Fig 8 describes the dynamic version of Moody Scheduling, whilst Fig 9 details the steps carried out in its adaptive version.

## 8 Experimental evaluation

We have used a software-based TLS framework called ATLaS [50] in order to execute in parallel five different applications with irregular loops that are not parallelizable at compile time, with and without dependencies among iterations. We first introduce ATLaS’ main features, and we then show what happens when we speculatively execute these applications in the ATLaS framework using different scheduling policies.

### 8.1 The ATLaS TLS framework

ATLaS is a complete TLS framework that allows the use of software-based speculative techniques to parallelize loops whose parallelization at compile time is considered unsafe. The ATLaS framework consists of two parts. The first is a library that manages speculative execution at runtime [4, 11]. Using this library, the loop is divided into chunks of iterations, whose size depends on the scheduling policy being used. The execution of each chunk is assigned to a different thread. The library is based on an aggressive sliding window, that consists of an array of slots which store the status of each running thread, and pointers to their versions of the speculative data. Commits are carried out in order from the non-speculative thread. Each time a commit operation is finished, the sliding window advances one position, allowing a new, most-speculative thread to start. This library uses eager conflict detection, checks for data dependence violations on every speculative store, and avoids synchronization whenever possible. In fact, the ATLaS framework only requires the use of a critical section to schedule the next chunk to an available thread, and to commit results once a chunk has successfully executed: Both speculative reads and writes were carefully designed to avoid the use of explicit synchronization operations. Communication among threads are carried out with the help of a shared memory space, carefully designed to allow concurrent reads and writes without incurring in race conditions, and using barriers which ensures sequential consistency when needed [4]. If a dependence violation is found, the library offers two different squashing policies [7]: To squash the offending thread and all its successors (a solution called *inclusive squashing*) or to squash just the offending thread and those successors that have consumed data generated by it (*exclusive squashing*).

The second part of the ATLaS framework is a GCC plugin that extends OpenMP with a new *speculative()* clause [51–53]. This extension allows the programmer to indicate that a loop should be speculatively parallelized, and to declare the set of variables whose accesses may potentially lead to a dependence violation. The use of variables labeled as speculative is then monitorized by the runtime library to prevent dependence violations.

In this work, we have extended the ATLaS framework to support different scheduling policies: FSC, Meseta, dynamic and adaptive Just-in-Time (in their different versions), and dynamic and adaptive Moody Scheduling. The study has been carried out using inclusive squashing. We have used this framework to parallelize five different applications, as described below.

The ATLaS framework and the benchmarks used can be freely downloaded from the ATLaS website [50].

### 8.2 Benchmark description

We used TREE [54] as one of the benchmarks because most of the runtime is spent to execute a loop which cannot be parallelized automatically with the standard tools.

In addition, we used the 2-Dimensional Convex Hull (2D-Hull), a Randomized Incremental algorithm due to Clarkson *et al*. [55]. The aim of this algorithm is to obtain the smallest enclosing convex polygon (convex hull) of a set of points. Since it depends completely in the points used as input data, it cannot be parallelized by state-of-the-art compilers. Thus, we performed our tests with three different input sets: A set of points defining a disc, a square and a Kuzmin distribution [56].

The 2D-MEC [57] tries to find the minimum enclosing circle containing a given set of points in the plane. As the result is computed incrementally, depending on input data, the application cannot be parallelized without the use of a speculative approach. However, in this case, a dependence violation requires to recalculate the whole solution, affecting severely the performance.

Finally, we used the Delaunay triangulation [58] of a two-dimensional set of points with an input set of 100K points. Table 2 describes briefly the characteristics of each application considered.

**Table 2:** Characteristics of the algorithms and input sizes used.

Algorithm	Input set description	Loop parallelized	Loop time as % of total time	Iterations per invocation	% of dependence violations	FSC chunk size used (iterations)
TREE	Off-axis parab. collision	accel_10	94	4 096	0	100
2D-Hull	Kuzmin, 10M points	Main loop	99	9 999 997	0.0008	11 000
2D-Hull	Square, 10M points	Main loop	99	9 999 997	0.0032	3 000
2D-Hull	Disc, 10M points	Main loop	99	9 999 997	0.021	1 250
2D-MEC	Disc, 10M points	Inner loop	99	Changes dynamically	0.009	1 800
Delaunay	100K points	Main loop	99	95 000	0.5	2

### 8.3 Experimental methodology

Experiments were carried out on a 64-cores server, equipped with four 16-core AMD Opteron 6376 processors at 2.3GHz and 256GB of RAM, which runs CentOS Linux 7.0.1406 with dynamic frequency scaling disabled (preliminary results presented at EuroPar conference [46] were obtained in the same machine but without disabling frequency scaling). All threads had exclusive access to the processors during the execution of the experiments, and we used wall-clock times in our measurements. For each application and scheduling policy, we have run a set of experiments varying the number of threads from two to 64, always in the same order. Each set was repeated three times, obtaining variations in the execution times smaller than 1%. The average times obtained have been used to calculate the corresponding speedups. Applications were compiled with the gcc compiler and -O4 -fopenmp flags. Times shown below represent the time spent in the execution of the main loop of the application. The time needed to read the input set and the time needed to output the results have not been taken into account. All the speculative versions are compared with their original, sequential version in the same architecture.

### 8.4 Performance

**Fig 10:**
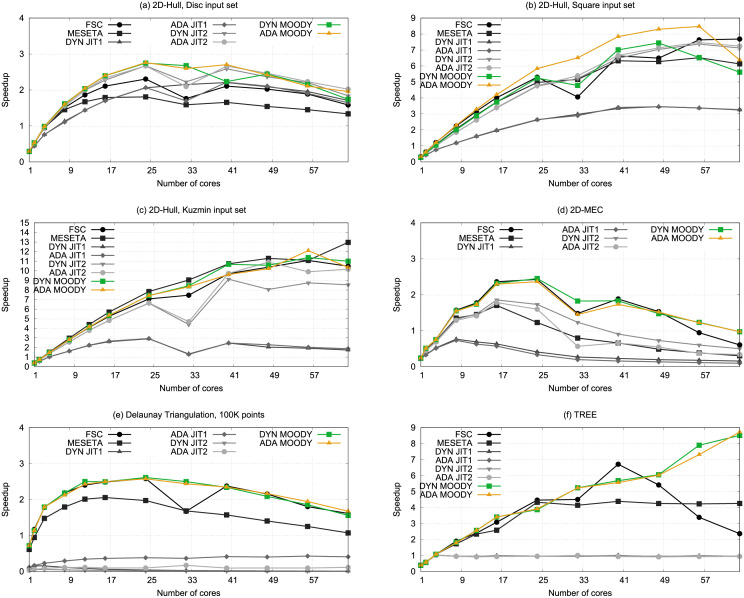
Performance comparison for 2D-Hull with disc, square, and kuzmin input sets, and 2D-MEC, delaunay, and TREE.

Fig 10 depicts the results obtained in the ATLaS speculative parallelization framework [52] for the applications described, using the previously reviewed scheduling mechanisms: Fixed-Size Chunking (FSC); Meseta; Dynamic (DYN) and Adaptive (ADA) Just-In-Time, using Eqs 1 and 2; and the new Dynamic and Adaptive Moody Scheduling methods.

The use of FSC requires a particular chunk size to be chosen. To be as fair as possible with this method, we have searched for the chunk size that delivers the best performance for each benchmark and input set used. This search required more than 20 dry-runs per application. These dry-runs used the same benchmark input that was used for the evaluation of FSC. Regarding the tuning parameters for the remaining scheduling methods, for Moody Scheduling we defined accMeanH = 2, $\beta =\frac{\pi }{4}$, and a value for *h* (the size of the window to be considered) equal to twice the number of processors for all applications. In regard to *α*, the values vary in the following interval $\alpha \in (\frac{\pi }{20},\frac{\pi }{6})$ depending on the dependence violations that may likely arise at runtime. Concerning the Meseta scheduling, we implemented a trapezoidal approach [20]. To test our benchmarks, we took a 10% of the total amount of iterations for the first zone, another 10% for the final zone, and the rest for the ‘tableland’. Fixed values chosen for the middle zone for this solution also required several executions to be optimized.

In relation to 2D-Hull (Fig 10(a)–10(c)), the Moody scheduling methods lead to a better performance than the best FSC for the Disc and Square input sets. For the Disc input set, the Adaptive Moody version produces the highest speedup (2.76×) with 24 processors. For the Square input set, the Adaptive Moody version produces the highest speedup (8.47×) with 56 processors. Finally, the Kuzmin input set, an input that presents almost no dependence violations, are similar for the best FSC and both Moody versions.

The 2D Smallest Enclosing Circle (Fig 10(d)) also shows very similar performance figures for the best FSC and both Moody versions, with Just-in-Time scheduling using Eq 2 attaining 30 to 60% of the maximum speedup, and the remaining methods showed a poorer performance.

The Delaunay triangulation (Fig 10(e)) shows how Moody Scheduling achieves exactly the same speedup as the best FSC, with the exception of the experiment with 32 processors, where a particularly bad combination of dependence violations makes the FSC performance drop by one third. Meseta achieves a remarkable result, while the JIT scheduling performance drops to almost zero. The reason is that the best chunk size for this application is composed of very few iterations, and the JIT methods were shown to be too optimistic for this situation.

In the case of TREE (Fig 10(f)), which presents no dependence violations, results for Moody Scheduling are more consistent than those obtained for the remaining methods. The best performance obtained with FSC needed 40 processors. The performance obtained with Moody Scheduling, on the other hand, made use of all the available processors, as it continues growing together with the number of processors available, achieving its peak when all are used. It is worthwhile noting that, for TREE, both the Dynamic and Adaptive mechanisms are similar: There are no dependence violations and, therefore, the size for each new chunk is computed only once. We obtained the best performance in this benchmark (8.72×) with the Adaptive version of Moody Scheduling and 64 processors, with the Dynamic version of Moody Scheduling delivering very similar performance figures.

It is also interesting to see the behavior of FSC with TREE. The irregular loop in TREE has 4096 iterations. Using 40 threads and a chunk size of 100 iterations is a near-optimal distribution, because each thread receives around 1/40 part of the loop. The last chunk of 96 iterations is executed by the first thread that finishes its work, while the remaining threads are still committing their results. Using more threads only adds execution overheads, since the additional threads should be started and stopped even when they do not receive any task, negatively affecting performance. Finally, recall that the irregular loop of TREE does not present dependence violations. This explains why adaptive and dynamic approaches deliver similar performance figures. The results show that, even without dependence violations, our self-tuning mechanism leads to better results than FSC even though it adds calls to the Moody Scheduling function.

Finally, it is worthwile mentioning that Moody Scheduling led to good performances even without any tuning: The performance of FSC with chunk size 1 drops almost to zero (except for Delaunay, when the best chunk size for FSC is 2), while Moody Scheduling reaches a performance of 88.3% of the best FSC on geometric average even if we set the initial chunk size to 1. In regard to Dynamic or Adaptive approaches, we cannot affirm whether any of them are better or worse than the other because the results vary depending on the application. As a result, both versions are included in the ATLaS framework.

### 8.5 Scheduling costs

A frequent objection to the use of sophisticated scheduling methods is that they would impose additional overheads that might not compensate for the performance gains. In this section we examine the cost associated to the calculation of the following chunk size for each of the scheduling methods and benchmarks considered, and how this cost grows with the number of processors.

To understand the nature of these overheads, it is important to know how ATLaS works. Each time a thread (either speculative or non-speculative) finishes its work, it enters a critical section to perform different tasks. If the thread is non-speculative, it commits its own results, and it also commits (in order) the results calculated by all subsequent threads that have already finished their work. It then forwards the non-speculative pointer to the first thread that is still working, which becomes the new non-speculative thread. If, on the contrary, the thread that has entered the critical section is speculative, it simply changes the state of its associated data structures from RUNNING to DONE, indicating that these data are ready to be committed when the non-speculative thread finishes its work. At this point is where the thread inside the critical section calls the scheduling function to calculate the size of the following chunk to be executed, and leaves the critical section to start its speculative execution [4].

**Fig 11:**
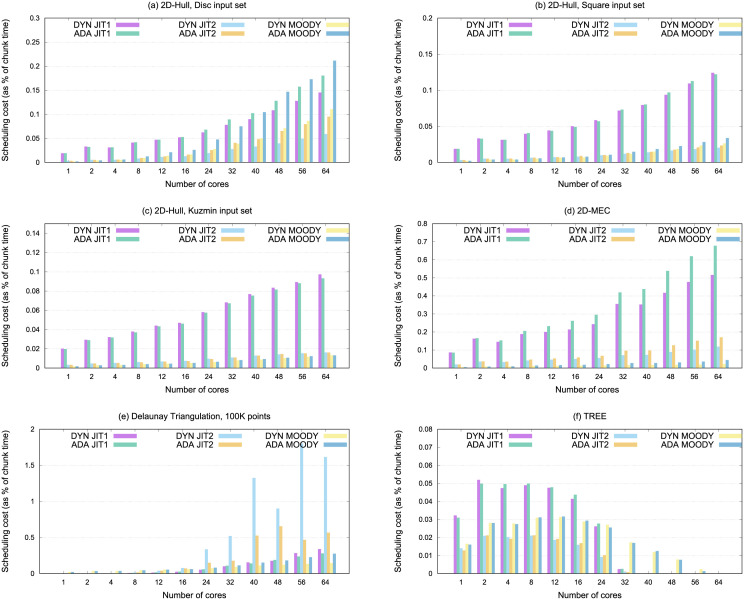
Time spent in the scheduling calculations for 2D-Hull with disc, square, and kuzmin input sets, and 2D-MEC, delaunay, and TREE.

Fig 11 details the costs associated with the calculation of the following chunk size in those approaches that define block sizes dynamically, namely JIT and Moody scheduling. In FSC, this cost is negligible, because the chunk size is fixed. In the case of Meseta, chunk sizes are just a function of the iteration number, not taking into account how the execution is progressing, and it is equally fast.

Our results show the scheduling cost as a percentage of the average cost of executing each chunk. To obtain the latter, we measured the accumulated execution time of all the chunks, dividing this time by the total number of chunks executed. Note that, due to the occurrence of dependence violations, some chunks have been executed multiple times before their results are committed. In these cases, dynamic variants only calculate the sizes of each new chunk once, while adaptive versions recalculate their sizes at each execution attempt of the new chunk.

In the case of the 2D-Hull with Disc input set (Fig 11(a)), the complex dependencies pattern makes the adaptive versions work harder; although the superior strategy of Moody Scheduling clearly compensates for the extra work in terms of performance (see Fig 10(a)). For the remaining benchmarks, the best choices carried out by the Moody strategy lead to fewer invocations to the scheduling function, which in turn leads to a better performance. Another interesting effect can be seen in the case of Delaunay Triangulation, a benchmark where the best chunk size is composed of very few iterations. In this case, as described above, using Just-in-Time scheduling with the formulation shown in Eq 2 turned out to be excessively optimistic.

Generally speaking, calculating chunk sizes dynamically or adaptively does not increase execution times. In most cases, iterations are dispatched with negligible costs (values lower than 0.05); therefore, it can be affirmed that runtime scheduling methods impose almost no additional charges regarding global performance.

Finally, an interesting question is how these scheduling methods can be applied to TLS solutions that take advantage of hardware support, such as Hardware Transactional Memory (HTM) extensions available in some machines (see e.g., [59, 60]). None of the scheduling methods described require large data structures to be maintained. FSC uses a fixed chunk size; Meseta calculates it dynamically using the iteration number; and both JIT and Moody Scheduling require the sizes of the last T chunks and their number of re-executions to be stored in order to perform the calculations of the following chunk size. Therefore, for all methods mentioned except FSC, hardware dedicated to calculating this size using this information would be needed.

## 9 Conclusions

The scheduling of iterations is a key factor that directly influences not only the dependence violations that occurred, but also the overall performance of parallel programs. In this paper we have explored the design space of TLS scheduling alternatives, reaching two main conclusions. First, that it is possible to achieve a better performance by scheduling threads using the information about the actual pattern of dependence violations available at runtime, despite the extra complexity and the added overheads. Second, as the Moody Scheduling method performance shows, it is possible to obtain the best speedups with a general method that does not rely on a-priori assumptions about the particular dependencies pattern of a given application, as it happens with methods that take advantage of the randomized nature of the algorithm being parallelized. Our present and future work include to explore other scheduling techniques that may be appropriate for particular domains.

All methods described in this paper are now part of the ATLaS TLS framework [52], which can be freely downloaded from the ATLaS website [50].
